# G-CSF Administration after the Intraosseous Infusion of Hypertonic Hydroxyethyl Starches Accelerating Wound Healing Combined with Hemorrhagic Shock

**DOI:** 10.1155/2016/5317630

**Published:** 2016-02-17

**Authors:** Hong Huang, Jiejie Liu, Haojie Hao, Chuan Tong, Dongdong Ti, Huiling Liu, Haijing Song, Chaoguang Jiang, Xiaobing Fu, Weidong Han

**Affiliations:** ^1^Institute of Basic Medicine, Chinese PLA General Hospital, Beijing 100853, China; ^2^Medical College of Nankai University, Tianjin 300071, China; ^3^Burns Institute, First Affiliated Hospital of Chinese PLA General Hospital, Beijing 100853, China

## Abstract

*Objective.* To evaluate the therapeutic effects of G-CSF administration after intraosseous (IO) resuscitation in hemorrhagic shock (HS) combined with cutaneous injury rats.* Methods.* The rats were randomly divided into four groups: (1) HS with resuscitation (blank), (2) HS with resuscitation + G-CSF (G-CSF, 200 *μ*g/kg body weight, subcutaneous injection), (3) HS with resuscitation + normal saline solution injection (normal saline), and (4) HS + G-CSF injection without resuscitation (Unres/G-CSF). To estimate the treatment effects, the vital signs of alteration were first evaluated, and then wound closure rates and homing of MSCs and EPCs to the wound skins and vasculogenesis were measured. Besides, inflammation and vasculogenesis related mRNA expressions were also examined.* Results.* IO infusion hypertonic hydroxyethyl starch (HHES) exhibited beneficial volume expansion roles and G-CSF administration accelerated wound healing 3 days ahead of other groups under hemorrhagic shock. Circulating and the homing of MSCs and EPCs at wound skins were significantly elevated at 6 h after G-CSF treatment. Inflammation was declined since 3 d while angiogenesis was more obvious in G-CSF treated group on day 9.* Conclusions.* These results suggested that the synergistical application of HHES and G-CSF has life-saving effects and is beneficial for improving wound healing in HS combined with cutaneous injury rats.

## 1. Introduction

Traumatic hemorrhagic shock (THS) is a major preventable cause of death. Combined injuries, such as hemorrhagic shock (HS) combined with cutaneous injury, are common in the battlefield and traffic accidents. Hypovolemia, hypoxia, and microcirculation disturbance resulted from massive blood loss, which aggravates the course of wound healing [1, 2]. Cutaneous injuries are prone to develop into nonhealing wounds if treated inappropriately or neglected. Consequently, a better understanding of wound healing in cases of hemorrhagic shock combined with cutaneous injuries is necessary.

In HS, immediately establishing the resuscitation route is crucial. Intraosseous (IO) infusion, an alternative way of resuscitation, is very reliable and fast under the situation of which vascular access has collapsed and the intravascular route could not be quickly accessed [3, 4]. The resuscitation fluids expanded the circulation volume through the intramedullary sinus and part of bone marrow stem cells (BMSCs) could egress into circulation. Meanwhile, urgent signals are released in order to spontaneously recruit stem cells into the injured tissues to participate in wound repair under stress circumstances [5]. In hemorrhagic shock combined with cutaneous injury, it has been unknown whether early resuscitation through IO access would benefit wound healing by establishing effective circulation.

Of those BMSCs, mesenchymal stem cells (MSCs) and endothelial progenitor cells (EPCs) are particularly critical in wound healing [6, 7]. GFP-labeled MSCs transfusion and bone marrow transplantation experiments confirmed that the exogenous MSCs were capable of promoting wound healing by enhancing the paracrine effects as well as multipotential differentiation abilities [8–11]. EPCs are mobilized to damaged tissues and significantly promote angiogenesis by increasing the secretion of angiogenic cytokines in burned patients and myocardial infarcted rabbits [12, 13]. Additionally, bone marrow derived stem/progenitor cells could be strongly mobilized into circulation by cytokines such as granulocyte-colony stimulating factor (G-CSF) [14, 15]. Mobilized MSCs are essential in wound healing in cases of severe skin injury such as those from ionizing radiation, full-thickness cutaneous wounds, and cerebral tissue by regulating a series of cytokines and growth factors to ameliorate the microenvironment in wound areas to promote wound healing [9, 16, 17]. Consequently, we hypothesized that G-CSF treatment could enhance the mobilization as well as recruitment of MSCs and EPCs, which is conducive to wound healing in HS combined with cutaneous injury.

This study investigated the effects of G-CSF administration on wound healing after IO infusion HHES in hemorrhagic shock combined with cutaneous injury in a rat model. The composite treatment benefited in high volume circulation expansion and accelerated wound healing. The results provide a potential therapeutic strategy for treating this type of combined injury.

## 2. Methods

### 2.1. Hemorrhagic Shock Combined with Cutaneous Injury Rat Model

The investigation followed the Guidelines for the Institutional Animal Care Committee of the PLA General Hospital. Male Sprague-Dawley (SD) rats (250–300 g) were obtained from the experimental animal department of the Chinese PLA General Hospital. The animals were housed under conventional environmental conditions at ambient temperature, with access to pellet rodent chow and water ad libitum.

The rats were anesthetized with sodium pentobarbital intraperitoneal injection (40 mg/kg) and supplement during the experiment. First, a full-thickness excision (3 cm in diameter) in the left dorsal skin was performed. Then, carotid artery and jugular vein were isolated from anterior neck regions. Then, the right carotid artery was cannulated and connected with a pressure monitor system (BIOPAC, USA) to monitor mean arterial pressure (MAP) and heart rate (HR) values. After that, the controlled hemorrhagic shock rat model was induced via 40% blood withdrawing from the carotid artery in 1 h. The blood was collected with heparin sodium to prevent clotting in sterile tubes for later reinfusion. Blood samples were collected from jugular vein for detecting hemoglobin (HGB) and the hematocrit (HCT) values before injury, after resuscitation for 60 min and 2 h. Then, blood was obtained at 3 d, 5 d, 9 d, 13 d, and 17 d after injury. The polyethylene catheter, syringes, and tubing were washed with heparin sodium (1,000 U/mL) before all the procedures.

### 2.2. Intraosseous Infusion with HHES and G-CSF Treatment

After a hypotensive period of 60 min, the resuscitation group was subjected to the small volume of HHES (4 mL/kg body weight, 7.2% NaCl/6% hydroxyethyl starch, Fresenius Company, Bad Homburg, Germany) IO infusion. The HHES was injected into the tibial shaft with fixation of the left knee of the host rat by a gentle, firm pressure. Then, 50% of the lost blood was reinfused. During the infusion, the vital signs were monitored continuously for 2 h. And the resuscitated rats were randomly divided into three groups. The experiment group was given G-CSF subcutaneously (200 *μ*g/kg, rhG-CSF, Kyowa Hakko Kirin Co., Ltd., Japan) for 3 consecutive days. And control groups received an equal dose of normal saline (normal saline group) or did not receive (blank group) separately. Meanwhile, a group of rats which did not undergo resuscitation would receive G-CSF subcutaneously (200 *μ*g/kg) for 3 consecutive days and as the resuscitation control group (Unres/G-CSF). During the animal experiment, the temperature was maintained at 35~37°C by a heating pad. When the skins samples were harvested from the rats, they were sacrificed then.

### 2.3. Immunofluorescence (IF) Staining

Skin tissues from the wound sites were harvested at 3 d or 9 d after injury for IF staining of the CD34, CD90 or VWF, and Ki67. The skin samples were immersed in 4% cold paraformaldehyde overnight followed by embedding in OCT (Tissue-Tek; Sakura Finetek, CA, USA) as a frozen section for later analysis. For the IF staining, the cryostat sections (5 *μ*m) were incubated with the primary antibodies overnight at 4°C. Those antibodies were as follows. Ki67 was used as a typical proliferative marker (1 : 500), VWF (1 : 400) was a marker of vascular endothelial cells that were purchased from Abcam (Cambridge, UK), and CD34 and CD90 (1 : 50) (Santa Cruz, CA, USA) were used to indicate the homing of BMSCs at the wound sites. Then, the sections conjugated with an Alexa Fluor 594-conjugated anti-mouse IgG (1 : 500) and the nuclei were stained with hoechst33342 (1 : 3000) (Sigma, St. Louis, USA). The signals were visualized by a laser scanning confocal microscope (Olympus, Japan) and the analysis was performed on a FV10-ASW 1.7 viewer.

### 2.4. Quantitative Real-Time PCR (qRT-PCR)

The RNA samples were prepared from the wound skins obtained at 3, 5, 7, 9, 13, and 17 d after injury. Then, the cDNA of each sample was synthesized by a RevertAid*™* First Strand cDNA Synthesis Kit (Thermo Fisher Scientific) from 2 *μ*g of RNA. The relative mRNA expression was determined by qRT-PCR according to the THUNDERBIRD*™* SYBR qPCR Mix (TOYOBO, Japan) in a total 30 *μ*L volume. Next, qRT-PCR was performed on ABI PRISM 7500 (Applied Biosystems, CA, USA). The primer sequences are described in Table 1. Each cycle consists of activation at 95°C for 10 min, 40 cycles of denaturation at 94°C for 30 s, annealing at 55°C for 30 s, and extension at 72°C for 30 s to the platform stage, followed by 72°C for 10 min. Each sample was performed in triplicate. *β*-actin was chosen as the internal control gene. The relative gene expression was calculated using ABI PRISM 7500 version 2.0.6 software (Applied Biosystems, CA, USA) with the 2^−ΔΔCt^ method.

### 2.5. Flow Cytometry

The peripheral blood samples from the rats were obtained at normal state (0 h), 6 h, 3 d, 5 d, and 7 d after injury. Then, the species were lysate-treated and incubated with antibodies at room temperature for 20 min, washed, and finally suspended in PBS. Antibody CD29 Alexa Fluor®647, CD90-PerCP, CD31-PE, isotype antibody controls Alexa Fluor 647 Hamster IgM, *λ*1 isotype control, PerCP Mouse IgG1 *κ* isotype control, PE Mouse IgG1, *κ* isotype control, and rabbit IgG isotype control were purchased from BD Biosciences, CD34-Alexa Fluor 647 was purchased from Global Biotech, and CD45-FITC was purchased from Santa Cruz Biotechnology. The samples were collected and analyzed by flow cytometry (BD, Heidelberg, Germany).

### 2.6. Statistical Analysis

The data were expressed as the mean ± the standard error of the mean (SEM). The statistical comparisons were performed using paired Wilcoxon's test with SPSS 11.0 software (SPSS, Inc., USA). *P* values lower than 0.05 were considered statistically significant.

## 3. Results

### 3.1. IO Infusion HHES Restored the Vital Signs in Hemorrhagic Shock Combined with Cutaneous Injury Rats

In an effort to estimate the effect of early resuscitation by IO infusion HHES, 40% blood loss HS combined with cutaneous injury in a rat model was established. At completion of the 40% bloodshed, MAP significantly decreased to less than 40 mmHg, and the heart rate increased higher than 430 beats/min. After the 2 h resuscitation, all the rats survived, and the MAP and HR were restored to some extent (Table 2). However, all the groups underwent a decrease on HGB and HCT values after shock and resuscitation in 2 h. These data were consistent with previous results showing that early HHES resuscitation could effectively expand the blood volume. Considering that successive 3-day use of G-CSF may influence the hemodynamic parameters, we also detect the HGB and HCT values until wound healing (Table 3). The results suggested that 200 *μ*g/kg body weights for 3 days would not result in anemia or severe hematologic disorder whether receiving resuscitation or not.

### 3.2. G-CSF with HHES Elevated Circulating EPCs and MSCs

**Figure 1:**
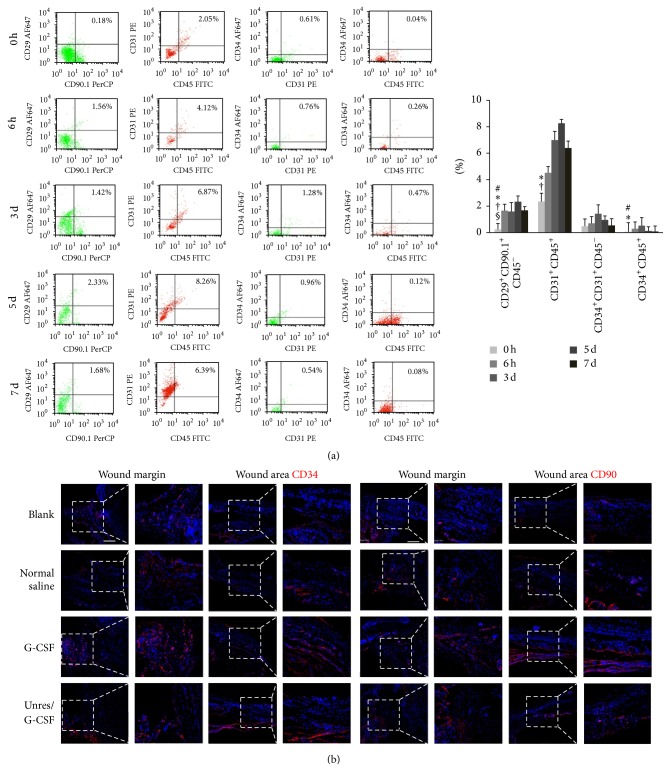
Elevated MSCs and EPCs anticipated wound healing in the hemorrhagic shock rats. (a) Representative density plots of the alterations of the BMSC distribution in the circulating blood at normal state (0 h), 6 h, 3 d, 5 d, and 7 d after G-CSF mobilization and HHES resuscitation. The typical cell surface markers represented as MSCs (CD45^−^CD29^+^CD90^+^), HSCs (CD45^+^CD31^+^ or CD45^+^CD34^+^), and EPCs (CD45^−^CD31^+^CD34^+^). ^#^ *P* < 0.05 versus the 6 h values; ^*∗*^ *P* < 0.05 versus 3 d values; ^†^ *P* < 0.05 versus 5 d values; ^§^ *P* < 0.05 versus 7 d values. (b) Representative images showed that CD34 (left, red) and CD90 (right, red) positive cells located both at the wound margins and wound areas at 6 h. Scale bar = 200 *μ*m. Each experiment was repeated three times and typical pictures were shown.

To confirm the mobilization of BMSCs during HS development, we examined the alterations of MSCs (CD45^−^CD29^+^CD90^+^), HSCs (CD45^+^CD31^+^ or CD45^+^CD34^+^), and EPCs (CD45^−^CD31^+^CD34^+^) in peripheral blood at 6 h and 3 d, 5 d, and 7 d by flow cytometry (FCM) (Figure 1(a)). According to the FCM results, MSCs, HSCs, and EPCs were significantly elevated in circulation compared to the normal state both at 6 h and 3 d after trauma. But the CD45^+^CD34^+^ and CD45^−^CD31^+^CD34^+^ subpopulations were declined at 5 d and dropped to the normal state at 7 d, while the CD45^−^CD29^+^CD90^+^ and CD45^+^CD31^+^ subpopulations were peaked at 5 d and then went down at 7 d. We further detected the recruitment of MSCs and EPCs at the wound sites by IF staining at 6 h after injury. The results suggested that the CD90^+^ and CD34^+^ cells were found as early as 6 h both at wound margin and wound areas (Figure 1(b)). The above results demonstrated that IO infusion HHES followed by G-CSF administration stimulated MSCs and EPCs egress into circulation and enhanced recruitment at the wound skins.

### 3.3. G-CSF Accelerated Wound Repair after HHES Infusion in Hemorrhagic Shock Combined with Cutaneous Injury in a Rat Model

**Figure 2:**
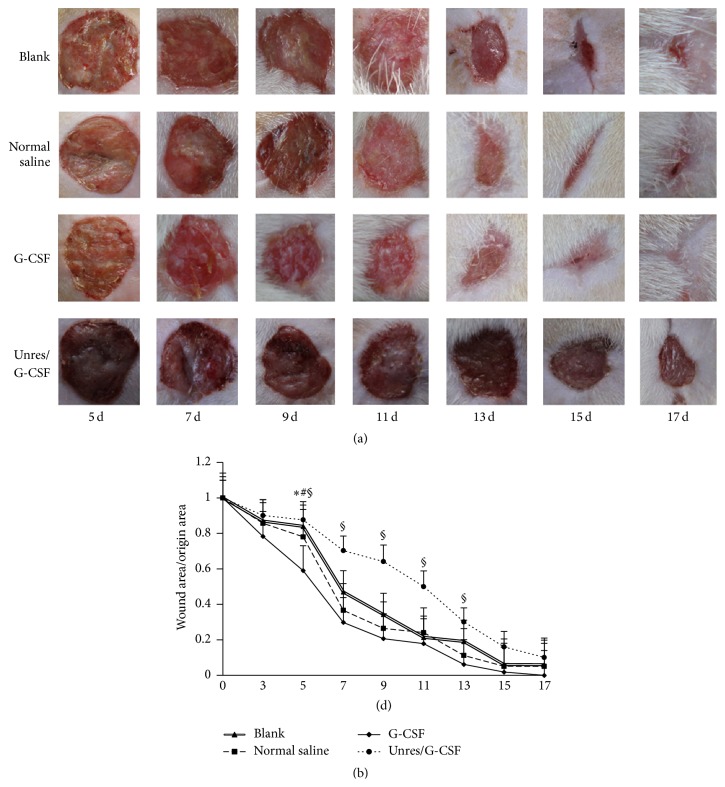
HHES accompanied G-CSF accelerated wound healing in hemorrhagic shock rats. (a) The morphology of the wound sites during the wound closure. (b) The relative healing rates during wound healing, the data are shown as the means ± SD; ^*∗*^ *P* < 0.05 versus the blank in the same group; ^#^ *P* < 0.05 versus normal saline in the same group. ^§^ *P* < 0.05 versus Unres/G-CSF group. Unresuscitated rats with G-CSF (Unres/G-CSF), induction of hemorrhagic shock without resuscitation but with G-CSF injection.

To illuminate the effects of G-CSF on wound closure after HHES resuscitation, the healing time of each group was compared. The results showed that G-CSF accelerated wound healing with the wound closure occurring 3 days ahead of the control groups (Figures 2(a) and 2(b)). According to the data on day 5, the G-CSF group had an obviously faster wound healing rate than the normal saline group, blank group, and the Unres/G-CSF group (41 ± 7%, 22 ± 5%, 19 ± 9%, and 12 ± 4%, resp.). The Unres/G-CSF group had the lowest healing rate than the other 3 groups. This may result from the tissue hypoxia and lack of perfusion. These data suggested that G-CSF could accelerate wound healing after IO resuscitation in HS combined with cutaneous injury rats.

### 3.4. G-CSF Treatment after HHES Infusion Attenuated Inflammation Reaction and Promoted Angiogenesis in Wound Areas

**Figure 3:**
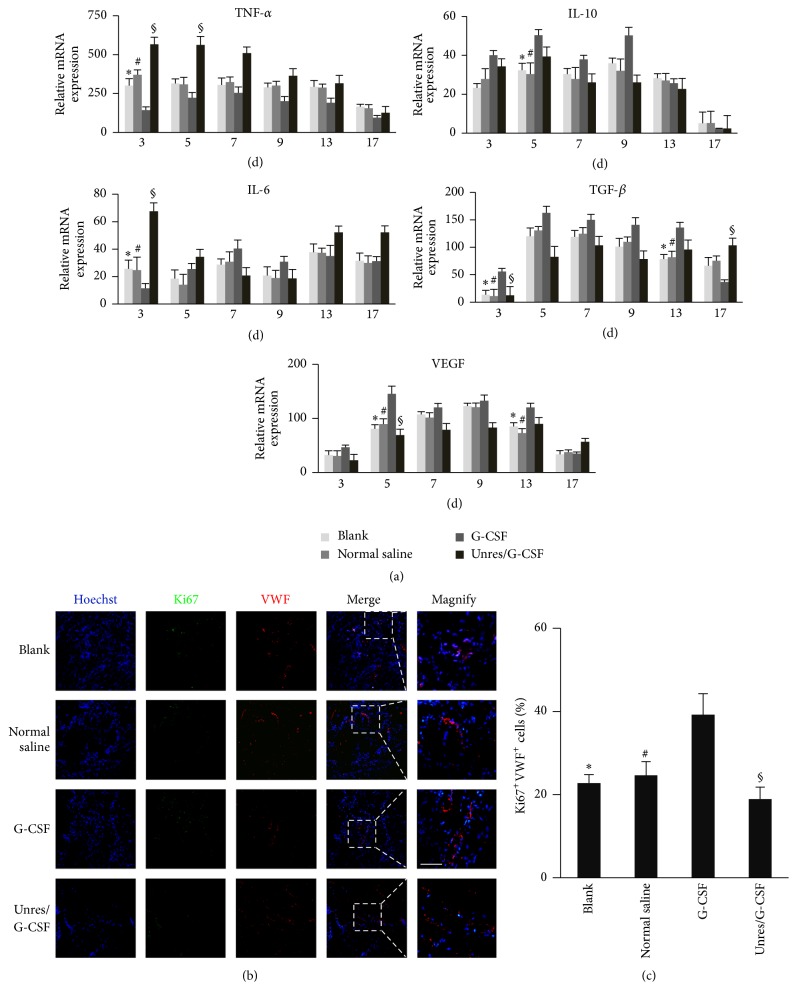
G-CSF combined with HHES promoted angiogenesis in the wound areas. (a) Typical cytokines relative expression of IL-6, IL-10, TGF-*β*, TNF-*α*, and VEGF mRNA during the wound healing. (b) VWF and Ki67 IF staining of the wound areas on day 9. Three-color fluorescent images of the vasculature in the frozen sections of the wound sites that were stained for Ki67 (green), VWF (red), and hoechst33342 (blue). (c) The mean percentages of the proliferative blood vessels coexpressing VWF (red) and Ki67 (green) at the wound sites. The data are shown as the means ± SEM. ^*∗*^ *P* < 0.05 versus the blank in the same group; ^#^ *P* < 0.05 versus normal saline in the same group. ^§^ *P* < 0.05 versus Unres/G-CSF in the same group. Unresuscitated rats with G-CSF (Unres/G-CSF), induction of hemorrhagic shock without resuscitation but with G-CSF injection. The white dashed frame indicated magnification typical VWF/Ki67 double staining areas. Scale bar = 50 *μ*m.

Since the inflammatory and angiogenesis related factors were important to wound healing, we further examined the relative mRNA expression of IL-10, IL-6, VEGF, TGF-*β*, and TNF-*α* in wound sites by qRT-PCR (Figure 3(a)). The semiquantification implied that the inflammatory factor TNF-*α* was significantly downregulated while the anti-inflammatory factor IL-10 was upregulated from an early stage to a greater degree in the G-CSF group than in the other groups. As to IL-6, a duel-regulated cytokine was expressed less in the G-CSF group at 3 d and increasingly elevated from 5 d. The angiogenesis related factors, such as VEGF and TGF-*β*, were more highly expressed in G-CSF group till 13 d. Moreover, we focused on angiogenesis during wound healing by IF staining of VWF and Ki67 on day 9. The results demonstrated that the G-CSF treatment increased the VWF positive cells and Ki67/VWF double-positive cells compared with the control groups on 9 d (Figures 3(b) and 3(c)). Collectively, these results demonstrated that G-CSF treatment after IO resuscitation attenuated inflammation and elevated angiogenic factors expression, which further promoting angiogenesis.

## 4. Discussion

Hemorrhagic shock combined with cutaneous injury is common in the battlefield and surgical operations [18]. And wound healing is easily delayed by severe HS. In this study, early IO infusion of small bolus HHES was effective in increasing circulation volume and generating a positive effect on life-threatening HS combined with cutaneous injury. Subsequent administration of G-CSF was shown to promote wound healing under HS in a rat model. It was shown that HHES together with G-CSF could induce BMSCs mobilization and attenuate inflammatory reaction and facilitated angiogenesis, which contributed to accelerating wound closure.

At the occurrence of HS, immediate therapy to stop the bleeding and establish a resuscitation route is necessary. It has been demonstrated that IO infusion had an advantage over IV infusion in the first insertion success rate and lower complication rate in prehospital emergency treatment of hypovolemia patients [19–21]. In view of the importance of early fluid treatment to recover tissue and cellular perfusion, limited fluid resuscitation causes a mild alteration of the immune system and a reduction in complications instead of aggressive fluid resuscitation in THS [22]. Several studies suggested that HHES had advantage of high volume expansion efficiency with smaller doses and prevention of side effects such as acute pulmonary edema and neurosurgical procedures [23, 24]. We established a rat model of 40% blood loss with massive skin excision injury, and early IO infusion provided reliable resuscitation effects to improve circulation. After resuscitation, all the rats were alive and the basic vital signs tended towards stability. These results showed that early infusion HHES could be an efficient method of resuscitation by both expanding volume and preventing further deterioration.

Wound healing has been easily underestimated during HS combined with cutaneous injury. Simple disinfection and dressing of the wound are insufficient and lead to secondary wounds and scarring [2]. Cutaneous injury spontaneously triggers signals to promote activated and proliferated stem cells to migrate towards wound regions and mediate wound repair. However, the circulation failure from HS was hypothesized to hinder the mobilization and migration of MSCs and EPCs to the wound tissues. Meanwhile, early-stage BMSCs recruitment appears to be critical in the wound repair process. It had been demonstrated that early mobilizing BMSCs in the acute inflammatory stage would play important roles mainly by ameliorating inflammation and enhancing angiogenesis during wound repair [5, 25]. As G-CSF mobilized the bone marrow derived MSCs and EPCs, the data implied that the augmentation of MSCs and EPCs in both circulating and wound sites in turn would benefit wound healing partly by exerting paracrine effects to the wound areas. It had been also reported that mobilized MSCs could differentiate into cells participating the skin reconstruction at a relative low percentage [9].

G-CSF could be produced by several tissues and plays well-known roles in mobilizing BMSCs. There was evidence that showed that MSCs and EPCs were components of the bone marrow pool that actively participated in the tissue recovery [26]. EPCs were previously reported to differentiate into functional endothelia cells (ECs) that assembled microvessels and macrovessels which facilitated carrying nutrient capacity and oxygen transportation [27]. Coculture of MSC conditioned medium and ECs showed that MSCs modulated the proangiogenic protein expression, such as VEGF, to promote its angiogenic potential [10]. As the mRNA expression results in the G-CSF treated group showed, the essential cytokines such as VEGF and IL6 were augmented, and they played critical roles in angiogenesis at the inflammation and proliferative stage. The upregulated IL-10 mRNA and downregulated TNF-*α* were hypothesized to be associated with the anti-inflammatory effects of HHES and MSCs [5]. The related genes were modulated early to attenuate the inflammation stage as well as promoted angiogenesis. The Ki67^+^/VWF^+^ cells detected by IF indicated that the proliferative ECs and the enhanced vasculature contributed to angiogenesis, which benefited wound healing. However, the differentiation capacities of mobilized MSCs and EPCs at the wound skin were not examined, which need to be paid attention to in future studies.

This study evaluated the potential role of G-CSF in wound closure after IO resuscitation in hemorrhagic shock combined with cutaneous injury. Our results highlighted that the combined treatment would have a therapeutic effect in suppressing the inflammatory response and vigorously promoting angiogenesis, which indicated that the treatment is effective in the rescue of casualties resulting from war and traffic accidents. These results provide a basis for future investigation of hemorrhagic shock and demonstrate the efficacy of G-CSF as a potent complementary solution for wound healing.

## Figures and Tables

**Table 1:** Primers had been used in qRT-PCR.

Primers	Temperature	Length	Sequences
IL6	55°C	163 bp	Forward CCGGAGAGGAGACTTCACAG
			Reverse GACAGTGCATCATCGCTGTTC

IL10	55°C	191 bp	Forward TGGCCCAGAAATCAAGGAGC
			Reverse GAAGATGTCAAACTCATTCATGC

TGF *β*	55°C	154 bp	Forward ATACGCCTGAGTGGCTGTCT
			Reverse TTGGGACTGATCCCATTGAT

TNF *α*	55°C	172 bp	Forward TCCGCAGATACCTGGAACTC
			Reverse CTCAGATCCTCCCCATTCAA

VEGF	55°C	172 bp	Forward GCCCATGAAGTGGTGAAGTT
			Reverse ACTCCAGGGCTTCATCATTG

*β*-actin	56°C	580 bp	Forward AGAGGGAAATCGTGCGTGAC
			Reverse CATCTGCTGGAAGGTGGACA

**Table 2:** The vital signs after resuscitation of hemorrhagic shock with cutaneous injured rats.

Variables	Group	Baseline	HS	60 min	120 min
MAP (mm Hg)	Blank	131 ± 7	37 ± 16^**∗**^	67 ± 23^**∗**^	88 ± 24
	Normal saline	122 ± 6	39 ± 13^**∗**^	61 ± 25^**∗**^	78 ± 27^**∗**^
	G-CSF	120 ± 9	35 ± 14^**∗**^	70 ± 29^**∗**^	89 ± 17
	Unres/G-CSF	125 ± 6	37 ± 11^**∗**^	36 ± 13^**∗**^	34 ± 14^**∗**^

HR (beat/min)	Blank	382 ± 7	435 ± 26^**∗**^	429 ± 21^**∗**^	411 ± 20
	Normal saline	367 ± 10	451 ± 19^**∗**^	438 ± 20^**∗**^	419 ± 19
	G-CSF	371 ± 11	448 ± 22^**∗**^	435 ± 26^**∗**^	415 ± 23
	Unres/G-CSF	385 ± 9	440 ± 27^**∗**^	406 ± 29^**∗**^	402 ± 18

HGB (g/L)	Blank	97.9 ± 19.2	91.5 ± 15.6	81.4 ± 14.5	76.2 ± 16.8^**∗**^
	Normal saline	92.4 ± 23.7	88.4 ± 16.8	84.6 ± 11.3	71.4 ± 14.8^**∗**^
	G-CSF	101.6 ± 21.2	90.2 ± 17.5	79.8 ± 19.1^**∗**^	68.3 ± 17.6^**∗**^
	Unres/G-CSF	105.1 ± 12.2	93.2 ± 16.1	80.8 ± 13.2^**∗**^	78.3 ± 14.2^**∗**^

HCT (%)	Blank	37 ± 5	29 ± 7	24 ± 2^**∗**^	21 ± 2^**∗**^
	Normal saline	34 ± 6	26 ± 4	23 ± 3^**∗**^	22 ± 1^**∗**^
	G-CSF	38 ± 8	24 ± 9^**∗**^	22 ± 2^**∗**^	21 ± 1^**∗**^
	Unres/G-CSF	39 ± 4	25 ± 8^**∗**^	21 ± 3^**∗**^	20 ± 2^**∗**^

Data are mean ± SD. MAP, mean arterial pressure; HR, heart rate; HGB, hemoglobin; HCT, hematocrit. Unres/G-CSF, shocked rats with G-CSF injection without resuscitation.

^*∗*^ *P* < 0.05 versus the values measured before shock and cutaneous injury.

**Table 3:** The hemoglobin and hematocrit alterations during wound healing in hemorrhagic shock with cutaneous injured rats.

Variables	Group	3 d	5 d	9 d	13 d	17 d
HGB (g/L)	Blank	78.4 ± 10.3	92.6 ± 9.1	96.4 ± 10.3	104.5 ± 14	98.6 ± 11.3
	Normal saline	83.1 ± 9.5	96.2 ± 6.8	98 ± 10.6	94.8 ± 12.8	101.5 ± 13.7
	G-CSF	76 ± 13.1	97.3 ± 12.2	102.4 ± 9.7	100.3 ± 11.1	103.5 ± 9.8
	Unres/G-CSF	72.7 ± 7.5	94.9 ± 7.1	94.1 ± 11.2	98.2 ± 17.5	99.5 ± 15.9

HCT (%)	Blank	26 ± 5	29 ± 5	29 ± 2	30 ± 4	36 ± 1
	Normal saline	25 ± 5	30 ± 3	31 ± 1	32 ± 2	34 ± 1
	G-CSF	29 ± 4	30 ± 4	32 ± 2	34 ± 3	32 ± 3
	Unres/G-CSF	28 ± 4	29 ± 3	30 ± 5	30 ± 4	31 ± 5

Data are mean ± SD; HGB, hemoglobin; HCT, hematocrit. Unres/G-CSF, shocked rats with G-CSF injection without resuscitation. There are no significant differences among groups.
